# An octa­nuclear zinc(II) complex with 6,6′-dihydr­oxy-2,2′-[1,2-phenyl­enebis(nitrilo­methyl­idyne)]diphenol

**DOI:** 10.1107/S1600536808017340

**Published:** 2008-06-13

**Authors:** Naser Eltaher Eltayeb, Siang Guan Teoh, Suchada Chantrapromma, Hoong-Kun Fun, Rohana Adnan

**Affiliations:** a School of Chemical Science, Universiti Sains Malaysia, 11800 USM, Penang, Malaysia; b Crystal Materials Research Unit, Department of Chemistry, Faculty of Science, Prince of Songkla University, Hat-Yai, Songkhla 90112, Thailand; c X-ray Crystallography Unit, School of Physics, Universiti Sains Malaysia, 11800 USM, Penang, Malaysia

## Abstract

The asymmetric unit of the title compound, tetra­aqua­tetrakis­{μ_3_-6,6′-di­oxido-2,2′-[1,2-phenyl­enebis(nitrilo­methyl­idyne)]diphenolato}octa­zinc(II) dimethyl sulfoxide tetra­solvate dihydrate, [Zn_8_(C_20_H_12_N_2_O_4_)_4_(H_2_O)_4_]·4C_2_H_6_OS·2H_2_O, contains one quarter of a Zn^II^ octa­nuclear complex with 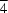 symmetry, one dimethyl sulfoxide mol­ecule and one half of a water mol­ecule which lies on a twofold rotation axis. The Zn^II^ atoms of the octa­nuclear complex have two different five-coordinate environments, *viz*. ZnN_2_O_3_ and ZnO_5_. All eight Zn^II^ centers adopt a distorted square-pyramidal coordination; four Zn^II^ ions have the N_2_O_2_ tetra­dentate Schiff base ligand bound in a basal plane and the coordinated water mol­ecule occupying the apical site, while the remaing four Zn^II^ ions are bound by five O atoms from three Schiff base ligands. In the crystal structure, Zn^II^ complex mol­ecules, coordinated and uncoord­inated water mol­ecules and dimethyl sulfoxide mol­ecules are linked *via* O—H⋯O and C—H⋯O hydrogen bonds and C—H⋯π inter­actions, forming a three-dimensional framework.

## Related literature

For related literatures on Schiff base Zn^II^ coordination complexes, see: Basak *et al.* 2007 ▶; Collinson & Fenton (1996 ▶); Pal *et al.* (2005 ▶); Tarafder *et al.* (2002 ▶). For related structures, see: Eltayeb *et al.* (2007*a* ▶,*b* ▶,*c* ▶). For bond-length data, see: Allen *et al.* (1987 ▶).
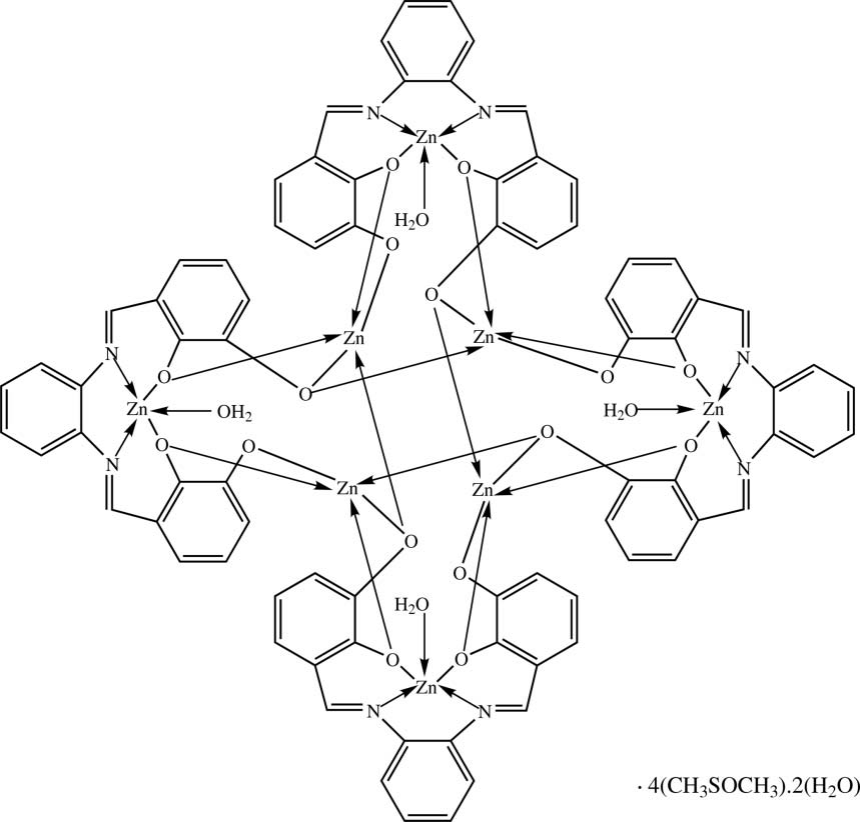


## Experimental

### 

#### Crystal data

[Zn_8_(C_20_H_12_N_2_O_4_)_4_(H_2_O)_4_]·4C_2_H_6_OS·2H_2_O
*M* *_r_* = 2321.03
Tetragonal, 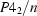

*a* = 18.1324 (3) Å
*c* = 13.3813 (3) Å
*V* = 4399.56 (14) Å^3^
*Z* = 2
Mo *K*α radiation
μ = 2.32 mm^−1^
*T* = 100.0 (1) K
0.57 × 0.13 × 0.10 mm

#### Data collection

Bruker SMART APEXII CCD area-detector diffractometer
Absorption correction: multi-scan (*SADABS*; Bruker, 2005 ▶) *T*_min_ = 0.352, *T*_max_ = 0.796
26700 measured reflections
5851 independent reflections
3700 reflections with *I* > 2σ(*I*)
*R*_int_ = 0.085

#### Refinement

*R*[*F* ^2^ > 2σ(*F* ^2^)] = 0.050
*wR*(*F* ^2^) = 0.138
*S* = 1.02
5851 reflections
314 parameters
6 restraints
H atoms treated by a mixture of independent and constrained refinement
Δρ_max_ = 0.67 e Å^−3^
Δρ_min_ = −1.34 e Å^−3^

### 

Data collection: *APEX2* (Bruker, 2005 ▶); cell refinement: *APEX2*; data reduction: *SAINT* (Bruker, 2005 ▶); program(s) used to solve structure: *SHELXTL* (Sheldrick, 2008 ▶); program(s) used to refine structure: *SHELXTL*; molecular graphics: *SHELXTL*; software used to prepare material for publication: *SHELXTL* and *PLATON* (Spek, 2003 ▶).

## Supplementary Material

Crystal structure: contains datablocks global, I. DOI: 10.1107/S1600536808017340/ci2611sup1.cif

Structure factors: contains datablocks I. DOI: 10.1107/S1600536808017340/ci2611Isup2.hkl

Additional supplementary materials:  crystallographic information; 3D view; checkCIF report

## Figures and Tables

**Table 1:** Hydrogen-bond geometry (Å, °)

*D*—H⋯*A*	*D*—H	H⋯*A*	*D*⋯*A*	*D*—H⋯*A*
O1*W*—H1*W*1⋯O4^i^	0.84 (4)	1.72 (4)	2.535 (4)	164 (5)
O2*W*—H1*W*2⋯O5	0.85 (8)	2.28 (9)	3.032 (4)	147 (4)
O1*W*—H2*W*1⋯O5	0.83 (4)	1.95 (4)	2.772 (5)	174 (4)
C3—H3*A*⋯O3^ii^	0.93	2.57	3.271 (5)	132
C21—H21*C*⋯O4^iii^	0.96	2.52	3.454 (7)	165
C21—H21*B*⋯*Cg*1^iv^	0.96	2.81	3.475 (6)	127

Symmetry codes: (i) 
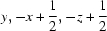
; (ii) 
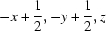
; (iii) 
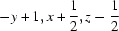
; (iv) 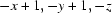.  *Cg*1 is the centroid of the C1–C6 ring.

**Fig. 1.**
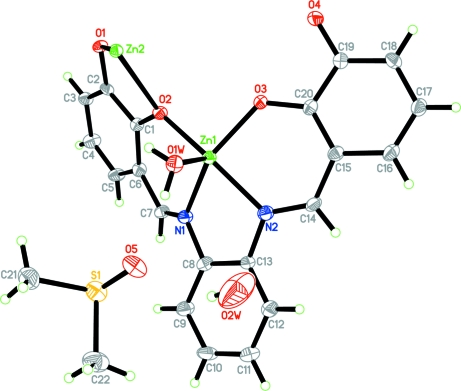
The asymmetric unit of the title compound. Displacement ellipsoids are drawn at the 50% probability level.

**Fig. 2.**
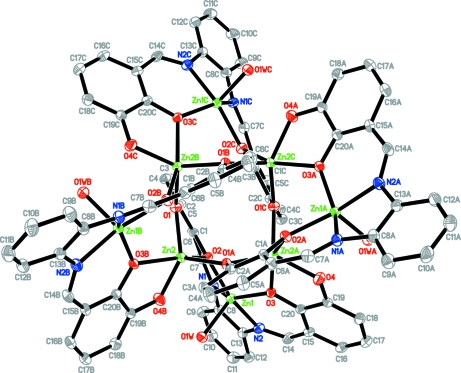
The molecular structure of the octanuclear complex, showing 50% probability displacement ellipsoids and the atomic numbering. H atoms of the ZnII complex, DMSO and solvated water molecules have been omitted for clarity. Symmetry codes: (A) 1/2 - y, x, 1/2 - z; (B) y, 1/2 - x, 1/2 - z; (C) 1/2 - x, 1/2 - y, z.

**Fig. 3.**
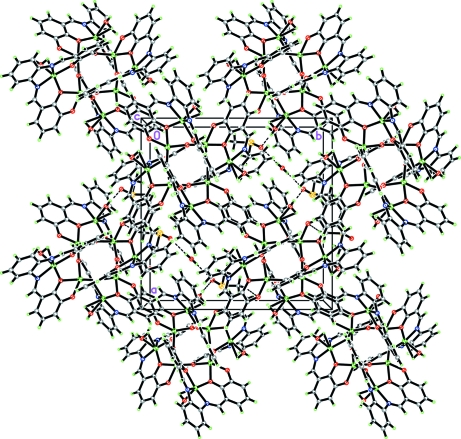
The crystal packing of the title compound, viewed along the c axis, showing sheets parallel to the ab plane. Hydrogen bonds are shown as dashed lines.

## supplementary crystallographic information

### Comment

There has been considerable interest in the synthesis of metal Schiff base complexes due to their coordination chemistry and applications (Basak *et al.*, 2007; Eltayeb *et al.*, 2007*a*,b,c; Pal *et al.*, 2005; Tarafder *et al.*, 2002). Zinc complexes with Schiff bases are important in biological systems and coordination chemistry (Collinson & Fenton, 1996; Tarafder *et al.*, 2002). Previously, we have reported crystal structures of Zn^II^ complexes with Schiff base ligands (Eltayeb *et al.*, 2007*a*,b,c). As a continuation of our research on Schiff base complexes, we report here the crystal structure of the title octanuclear Zn^II^ complex.

The asymmetric unit of the title compound (Fig. 1) contains one quarter of the Zn_8_(C_80_H_56_N_8_O_20_) complex, one dimethyl sulfoxide (C_2_H_6_OS) and one-half of a water molecule with its O atom lying on a twofold rotation axis. The other three quarters of the octanuclear complex molecule are generated by the fourfold axis. The Zn^II^ atoms of the octanuclear complex has two different five-coordination environments *viz*. ZnN_2_O_3_ and ZnO_5_ (Fig. 2). All eight Zn^II^ centers adopt a distorted square-pyramidal coordination in which four Zn^II^ ions (outer) (Zn1 and its three symmetry equivalents Zn1A, Zn1B and Zn1C) coordinate with the N_2_O_2_ tetradentate Schiff base ligand bounded in a basal plane and the coordinated water molecule occupying the apical site. The other four Zn^II^ ions (inner) (Zn2 and its three symmetry equivalents Zn2A, Zn2B and Zn2C) are coordinated with five O atoms from three Schiff base ligands (see Fig. 2). The Zn^II^ ions in each unit are connected by one µ-O (Zn1—O2—Zn2) atom (Fig. 1). The Zn—µ-O bond lengths are Zn1—O2 = 2.032 (3) and Zn2—O2 = 2.191 (3) Å. In the octanuclear cluster, the µ-O1 atoms are also in bridging positions, between the Zn^II^ ions (inner cavity) (Zn2—O1—Zn2B) with the Zn—µ-O distances of Zn2—O1 = 2.000 (3) Å and Zn2B—O1 = 1.981 (3) Å. The connections of the four inner Zn^II^ ions by bridging µ-O1 and its equivalents result in the formation of an eight membered ring (Zn2—O1—Zn2B—O1B—Zn2C—O1C—Zn2A—O1A), with the Zn···Zn contacts being 3.4878 (5) Å. The Schiff base ligand in the present complex is in an *umbrella* conformation with the dihedral angle between the two outer rings (C1—C6 and C15—C20) being 48.1 (2) °. In the octanuclear complex (Fig. 2), the four Schiff bases have their concave sides alternating up and down. The coordination geometry of the five-coordinate atoms Zn1 and Zn2 (and their equivalents) can be viewed as that of a slightly distorted square antiprism. Bond lengths and angles observed in the structure are in normal ranges (Allen *et al.*, 1987) and comparable with the related structures (Eltayeb *et al.*, 2007*a*,b,c).

In the crystal packing (Fig. 3), the Zn^II^ complex molecules, coordinated and free water molecules and dimethyl sulfoxide molecules are linked *via* O—H···O and C—H···O hydrogen bonds and C—H···π interactions involving the C1—C6 (centroid *Cg*1) ring (Table 1) forming a three-dimensional framework.

### Experimental

The title compound was synthesized by adding 2,3-dihydroxybenzaldehyde (0.552 g, 4 mmol) to a solution of *o*-phenylenediamine (0.216 g, 2 mmol) in ethanol 95% (20 ml). The mixture was refluxed with stirring for 30 min. Zinc chloride (0.544 g, 4 mmol) in ethanol (10 ml) was then added, followed by triethylamine (1.0 ml, 7.2 mmol). The mixture was stirred at room temperature for 3 h. The yellow precipitate obtained was washed with about 5 ml e thanol, dried, and then washed with copious amounts of diethylether. Orange single crystals of the title compound suitable for *X*-ray diffraction were formed after recrystallization in the dimethyl sulfoxide/ethanol (3:5 *v*/*v*) at room temperature after several days.

### Refinement

Water H atoms were found in the difference map and their positions were refined with a restrained geometry, with O—H = 0.84 (2) Å and H···H = 1.37 (2) Å. The remaining H atoms are placed in calculated positions with d(C—H) = 0.93 Å, *U*_iso_ = 1.2*U*_eq_(C) for CH and aromatic and 0.96 Å, *U*_iso_ = 1.5*U*_eq_(C) for CH_3_ atoms. A rotating group model was used for the methyl groups. The highest residual electron density peak is located at 0.36 Å from H22C and the deepest hole is located at 0.47 Å from S1.

### Crystal data

**Table:**

[Zn_8_(C_20_H_12_N_2_O_4_)_4_(H_2_O)_4_]·4C_2_H_6_OS·2H_2_O	*Z* = 2
*M**_r_* = 2321.03	*F*_000_ = 2360
Tetragonal, *P*4_2_/*n*	*D*_x_ = 1.752 Mg m^−^^3^
Hall symbol: -P 4bc	Mo *K*α radiation λ = 0.71073 Å
*a* = 18.1324 (3) Å	Cell parameters from 5851 reflections
*b* = 18.1324 (3) Å	θ = 2.9–29.0º
*c* = 13.3813 (3) Å	µ = 2.32 mm^−^^1^
α = 90º	*T* = 100.0 (1) K
β = 90º	Needle, orange
γ = 90º	0.57 × 0.13 × 0.10 mm
*V* = 4399.56 (14) Å^3^	

### Data collection

**Table:**

Bruker SMART APEXII CCD area-detector diffractometer	5851 independent reflections
Radiation source: fine-focus sealed tube	3700 reflections with *I* > 2σ(*I*)
Monochromator: graphite	*R*_int_ = 0.085
Detector resolution: 8.33 pixels mm^-1^	θ_max_ = 29.0º
*T* = 100.0(1) K	θ_min_ = 2.9º
ω scans	*h* = −24→11
Absorption correction: multi-scan(SADABS; Bruker, 2005)	*k* = −24→24
*T*_min_ = 0.352, *T*_max_ = 0.796	*l* = −18→18
26700 measured reflections	

### Refinement

**Table:**

Refinement on *F*^2^	Secondary atom site location: difference Fourier map
Least-squares matrix: full	Hydrogen site location: inferred from neighbouring sites
*R*[*F*^2^ > 2σ(*F*^2^)] = 0.050	H atoms treated by a mixture of independent and constrained refinement
*wR*(*F*^2^) = 0.138	*w* = 1/[σ^2^(*F*_o_^2^) + (0.0592*P*)^2^ + 6.834*P*] where *P* = (*F*_o_^2^ + 2*F*_c_^2^)/3
*S* = 1.02	(Δ/σ)_max_ = 0.001
5851 reflections	Δρ_max_ = 0.67 e Å^−^^3^
314 parameters	Δρ_min_ = −1.34 e Å^−^^3^
6 restraints	Extinction correction: none
Primary atom site location: structure-invariant direct methods	

### Special details

**Table:**

Experimental. The low-temparture data was collected with the Oxford Cyrosystem Cobra low-temperature attachment.
Geometry. All e.s.d.'s (except the e.s.d. in the dihedral angle between two l.s. planes) are estimated using the full covariance matrix. The cell e.s.d.'s are taken into account individually in the estimation of e.s.d.'s in distances, angles and torsion angles; correlations between e.s.d.'s in cell parameters are only used when they are defined by crystal symmetry. An approximate (isotropic) treatment of cell e.s.d.'s is used for estimating e.s.d.'s involving l.s. planes.
Refinement. Refinement of *F*^2^ against ALL reflections. The weighted *R*-factor *wR* and goodness of fit *S* are based on *F*^2^, conventional *R*-factors *R* are based on *F*, with *F* set to zero for negative *F*^2^. The threshold expression of *F*^2^ > σ(*F*^2^) is used only for calculating *R*-factors(gt) *etc*. and is not relevant to the choice of reflections for refinement. *R*-factors based on *F*^2^ are statistically about twice as large as those based on *F*, and *R*- factors based on ALL data will be even larger.

### Fractional atomic coordinates and isotropic or equivalent isotropic displacement parameters (Å^2^)

**Table:**

	*x*	*y*	*z*	*U*_iso_*/*U*_eq_	
Zn1	0.22916 (3)	0.46426 (3)	0.07015 (4)	0.01886 (13)	
Zn2	0.34013 (2)	0.35177 (2)	0.24587 (4)	0.01728 (13)	
O1	0.35359 (15)	0.25401 (15)	0.1791 (2)	0.0188 (6)	
O2	0.28402 (15)	0.36961 (15)	0.1029 (2)	0.0192 (6)	
O3	0.14565 (15)	0.43546 (15)	0.1585 (2)	0.0189 (6)	
O4	0.05238 (16)	0.38411 (16)	0.2903 (3)	0.0277 (7)	
N1	0.26712 (19)	0.45884 (18)	−0.0754 (3)	0.0210 (7)	
N2	0.1627 (2)	0.54198 (19)	−0.0038 (3)	0.0233 (8)	
C1	0.3139 (2)	0.3234 (2)	0.0380 (3)	0.0177 (8)	
C2	0.3494 (2)	0.2598 (2)	0.0780 (3)	0.0182 (8)	
C3	0.3795 (2)	0.2079 (2)	0.0141 (3)	0.0222 (9)	
H3A	0.4019	0.1661	0.0407	0.027*	
C4	0.3768 (3)	0.2170 (2)	−0.0899 (4)	0.0282 (10)	
H4A	0.3952	0.1805	−0.1320	0.034*	
C5	0.3471 (2)	0.2795 (2)	−0.1285 (3)	0.0248 (9)	
H5A	0.3470	0.2861	−0.1974	0.030*	
C6	0.3166 (2)	0.3342 (2)	−0.0672 (3)	0.0216 (9)	
C7	0.2939 (2)	0.4005 (2)	−0.1172 (3)	0.0231 (9)	
H7A	0.2990	0.4016	−0.1863	0.028*	
C8	0.2508 (2)	0.5228 (2)	−0.1328 (3)	0.0234 (9)	
C9	0.2866 (3)	0.5429 (3)	−0.2203 (4)	0.0296 (10)	
H9A	0.3238	0.5133	−0.2462	0.035*	
C10	0.2663 (3)	0.6077 (3)	−0.2689 (4)	0.0349 (12)	
H10A	0.2893	0.6207	−0.3285	0.042*	
C11	0.2123 (3)	0.6529 (3)	−0.2295 (4)	0.0326 (11)	
H11A	0.1990	0.6959	−0.2626	0.039*	
C12	0.1781 (3)	0.6342 (2)	−0.1410 (4)	0.0278 (10)	
H12A	0.1428	0.6655	−0.1138	0.033*	
C13	0.1960 (2)	0.5688 (2)	−0.0920 (3)	0.0218 (9)	
C14	0.0969 (2)	0.5594 (2)	0.0204 (4)	0.0250 (9)	
H14A	0.0727	0.5940	−0.0191	0.030*	
C15	0.0574 (2)	0.5294 (2)	0.1044 (3)	0.0223 (9)	
C16	−0.0143 (2)	0.5597 (2)	0.1196 (4)	0.0261 (10)	
H16A	−0.0298	0.5988	0.0797	0.031*	
C17	−0.0604 (2)	0.5332 (2)	0.1905 (4)	0.0269 (10)	
H17A	−0.1065	0.5546	0.1998	0.032*	
C18	−0.0388 (2)	0.4735 (2)	0.2496 (4)	0.0250 (9)	
H18A	−0.0707	0.4551	0.2978	0.030*	
C19	0.0295 (2)	0.4420 (2)	0.2369 (3)	0.0209 (9)	
C20	0.0805 (2)	0.4700 (2)	0.1638 (3)	0.0199 (9)	
S1	0.42019 (7)	0.60730 (7)	−0.06159 (11)	0.0376 (3)	
O5	0.3672 (2)	0.6353 (2)	0.0183 (3)	0.0436 (9)	
C21	0.5096 (3)	0.6047 (3)	−0.0076 (5)	0.0449 (14)	
H21A	0.5122	0.5650	0.0397	0.067*	
H21B	0.5192	0.6506	0.0258	0.067*	
H21C	0.5457	0.5972	−0.0591	0.067*	
C22	0.4357 (4)	0.6846 (3)	−0.1418 (5)	0.0504 (16)	
H22A	0.3913	0.6957	−0.1778	0.076*	
H22B	0.4744	0.6731	−0.1881	0.076*	
H22C	0.4498	0.7266	−0.1023	0.076*	
O1W	0.29110 (16)	0.53937 (16)	0.1423 (3)	0.0251 (7)	
H1W1	0.321 (2)	0.5050 (19)	0.154 (4)	0.038*	
H2W1	0.311 (2)	0.570 (2)	0.105 (3)	0.038*	
O2W	0.2500	0.7500	0.0625 (6)	0.104 (3)	
H1W2	0.278 (5)	0.724 (5)	0.026 (2)	0.156*	

### Atomic displacement parameters (Å^2^)

**Table:**

	*U*^11^	*U*^22^	*U*^33^	*U*^12^	*U*^13^	*U*^23^
Zn1	0.0204 (2)	0.0191 (2)	0.0171 (3)	0.00136 (18)	0.0007 (2)	0.0010 (2)
Zn2	0.0177 (2)	0.0191 (2)	0.0150 (2)	−0.00013 (18)	0.0004 (2)	−0.0005 (2)
O1	0.0245 (14)	0.0182 (13)	0.0139 (15)	0.0013 (11)	−0.0015 (13)	0.0006 (12)
O2	0.0229 (14)	0.0219 (14)	0.0128 (14)	0.0035 (11)	0.0021 (12)	−0.0020 (12)
O3	0.0182 (13)	0.0181 (13)	0.0205 (16)	0.0015 (10)	−0.0001 (12)	0.0022 (12)
O4	0.0263 (15)	0.0267 (15)	0.0302 (19)	0.0052 (12)	0.0069 (15)	0.0101 (15)
N1	0.0245 (17)	0.0203 (16)	0.0181 (19)	0.0016 (14)	0.0011 (16)	0.0018 (15)
N2	0.0261 (18)	0.0229 (17)	0.021 (2)	0.0033 (14)	0.0014 (17)	0.0014 (16)
C1	0.0195 (19)	0.0168 (18)	0.017 (2)	−0.0004 (15)	−0.0013 (17)	−0.0030 (17)
C2	0.0204 (18)	0.0192 (18)	0.015 (2)	0.0001 (15)	0.0052 (17)	−0.0002 (18)
C3	0.025 (2)	0.021 (2)	0.020 (2)	0.0031 (16)	0.0047 (19)	0.0000 (19)
C4	0.041 (3)	0.025 (2)	0.019 (2)	0.0074 (19)	0.003 (2)	−0.0046 (19)
C5	0.035 (2)	0.026 (2)	0.013 (2)	0.0031 (18)	0.0017 (19)	−0.0014 (19)
C6	0.023 (2)	0.023 (2)	0.019 (2)	0.0008 (16)	−0.0007 (19)	−0.0020 (19)
C7	0.024 (2)	0.030 (2)	0.015 (2)	0.0005 (17)	−0.0027 (19)	0.0018 (19)
C8	0.027 (2)	0.022 (2)	0.021 (2)	0.0005 (17)	−0.004 (2)	0.0010 (19)
C9	0.036 (3)	0.029 (2)	0.023 (2)	0.0043 (19)	0.002 (2)	0.004 (2)
C10	0.045 (3)	0.030 (2)	0.030 (3)	−0.002 (2)	0.007 (2)	0.011 (2)
C11	0.040 (3)	0.028 (2)	0.029 (3)	0.002 (2)	−0.002 (2)	0.009 (2)
C12	0.029 (2)	0.025 (2)	0.030 (3)	0.0036 (17)	0.001 (2)	0.002 (2)
C13	0.022 (2)	0.024 (2)	0.019 (2)	0.0000 (16)	−0.0007 (18)	0.0058 (18)
C14	0.029 (2)	0.024 (2)	0.022 (2)	0.0078 (17)	−0.001 (2)	0.0065 (19)
C15	0.026 (2)	0.0188 (19)	0.022 (2)	0.0027 (16)	−0.0011 (19)	−0.0004 (18)
C16	0.030 (2)	0.023 (2)	0.026 (3)	0.0071 (17)	−0.003 (2)	0.001 (2)
C17	0.027 (2)	0.027 (2)	0.026 (3)	0.0071 (18)	0.000 (2)	−0.001 (2)
C18	0.025 (2)	0.027 (2)	0.023 (2)	−0.0004 (17)	0.006 (2)	0.000 (2)
C19	0.0211 (19)	0.0185 (18)	0.023 (2)	−0.0011 (15)	−0.0010 (18)	−0.0030 (18)
C20	0.0210 (19)	0.0192 (19)	0.020 (2)	0.0004 (15)	−0.0038 (18)	−0.0025 (18)
S1	0.0380 (7)	0.0359 (7)	0.0389 (8)	−0.0037 (5)	0.0043 (6)	−0.0010 (6)
O5	0.044 (2)	0.045 (2)	0.042 (2)	−0.0087 (17)	0.0120 (19)	−0.0001 (19)
C21	0.038 (3)	0.057 (4)	0.039 (3)	−0.005 (2)	0.004 (3)	0.001 (3)
C22	0.062 (4)	0.042 (3)	0.048 (4)	0.007 (3)	0.021 (3)	0.005 (3)
O1W	0.0260 (16)	0.0203 (15)	0.0290 (19)	−0.0008 (12)	−0.0009 (14)	−0.0011 (14)
O2W	0.134 (8)	0.130 (8)	0.047 (5)	0.075 (6)	0.000	0.000

### Geometric parameters (Å, °)

**Table:**

Zn1—O3	1.990 (3)	C8—C9	1.387 (7)
Zn1—O1W	2.012 (3)	C8—C13	1.407 (6)
Zn1—O2	2.032 (3)	C9—C10	1.393 (6)
Zn1—N1	2.069 (4)	C9—H9A	0.93
Zn1—N2	2.101 (4)	C10—C11	1.381 (7)
Zn1—H1W1	2.13 (5)	C10—H10A	0.93
Zn2—O4^i^	1.973 (3)	C11—C12	1.379 (7)
Zn2—O1^ii^	1.981 (3)	C11—H11A	0.93
Zn2—O1	2.000 (3)	C12—C13	1.393 (6)
Zn2—O3^i^	2.152 (3)	C12—H12A	0.93
Zn2—O2	2.191 (3)	C14—C15	1.439 (6)
O1—C2	1.359 (5)	C14—H14A	0.93
O1—Zn2^i^	1.981 (3)	C15—C20	1.403 (6)
O2—C1	1.323 (5)	C15—C16	1.426 (6)
O3—C20	1.339 (5)	C16—C17	1.353 (7)
O3—Zn2^ii^	2.152 (3)	C16—H16A	0.93
O4—C19	1.336 (5)	C17—C18	1.396 (6)
O4—Zn2^ii^	1.973 (3)	C17—H17A	0.93
N1—C7	1.291 (5)	C18—C19	1.374 (6)
N1—C8	1.423 (5)	C18—H18A	0.93
N2—C14	1.277 (5)	C19—C20	1.439 (6)
N2—C13	1.412 (6)	S1—O5	1.525 (4)
C1—C6	1.421 (6)	S1—C21	1.775 (6)
C1—C2	1.425 (5)	S1—C22	1.787 (6)
C2—C3	1.384 (6)	C21—H21A	0.96
C3—C4	1.402 (6)	C21—H21B	0.96
C3—H3A	0.93	C21—H21C	0.96
C4—C5	1.356 (6)	C22—H22A	0.96
C4—H4A	0.93	C22—H22B	0.96
C5—C6	1.402 (6)	C22—H22C	0.96
C5—H5A	0.93	O1W—H1W1	0.837 (19)
C6—C7	1.436 (6)	O1W—H2W1	0.833 (19)
C7—H7A	0.93	O2W—H1W2	0.846 (14)
			
O3—Zn1—O1W	108.50 (13)	N1—C7—H7A	116.9
O3—Zn1—O2	91.31 (11)	C6—C7—H7A	116.9
O1W—Zn1—O2	101.24 (12)	C9—C8—C13	120.2 (4)
O3—Zn1—N1	143.11 (13)	C9—C8—N1	124.8 (4)
O1W—Zn1—N1	107.36 (14)	C13—C8—N1	114.9 (4)
O2—Zn1—N1	90.00 (12)	C8—C9—C10	119.5 (4)
O3—Zn1—N2	91.11 (13)	C8—C9—H9A	120.3
O1W—Zn1—N2	95.28 (13)	C10—C9—H9A	120.3
O2—Zn1—N2	161.59 (13)	C11—C10—C9	120.6 (5)
N1—Zn1—N2	77.25 (14)	C11—C10—H10A	119.7
O3—Zn1—H1W1	111.9 (13)	C9—C10—H10A	119.7
O1W—Zn1—H1W1	23.1 (7)	C12—C11—C10	120.1 (4)
O2—Zn1—H1W1	78.4 (6)	C12—C11—H11A	120.0
N1—Zn1—H1W1	104.5 (13)	C10—C11—H11A	120.0
N2—Zn1—H1W1	117.4 (6)	C11—C12—C13	120.6 (4)
O4^i^—Zn2—O1^ii^	117.28 (13)	C11—C12—H12A	119.7
O4^i^—Zn2—O1	128.45 (13)	C13—C12—H12A	119.7
O1^ii^—Zn2—O1	110.27 (16)	C12—C13—C8	119.1 (4)
O4^i^—Zn2—O3^i^	78.59 (12)	C12—C13—N2	126.0 (4)
O1^ii^—Zn2—O3^i^	112.98 (12)	C8—C13—N2	114.9 (4)
O1—Zn2—O3^i^	100.78 (11)	N2—C14—C15	124.8 (4)
O4^i^—Zn2—O2	80.99 (12)	N2—C14—H14A	117.6
O1^ii^—Zn2—O2	92.30 (11)	C15—C14—H14A	117.6
O1—Zn2—O2	78.31 (11)	C20—C15—C16	119.2 (4)
O3^i^—Zn2—O2	152.81 (11)	C20—C15—C14	125.7 (4)
C2—O1—Zn2^i^	124.8 (2)	C16—C15—C14	114.8 (4)
C2—O1—Zn2	111.7 (2)	C17—C16—C15	121.7 (4)
Zn2^i^—O1—Zn2	122.36 (15)	C17—C16—H16A	119.1
C1—O2—Zn1	126.4 (3)	C15—C16—H16A	119.1
C1—O2—Zn2	106.8 (2)	C16—C17—C18	120.0 (4)
Zn1—O2—Zn2	122.72 (14)	C16—C17—H17A	120.0
C20—O3—Zn1	125.4 (3)	C18—C17—H17A	120.0
C20—O3—Zn2^ii^	111.3 (2)	C19—C18—C17	120.4 (4)
Zn1—O3—Zn2^ii^	123.20 (13)	C19—C18—H18A	119.8
C19—O4—Zn2^ii^	117.4 (3)	C17—C18—H18A	119.8
C7—N1—C8	120.9 (4)	O4—C19—C18	122.7 (4)
C7—N1—Zn1	124.9 (3)	O4—C19—C20	116.2 (4)
C8—N1—Zn1	113.6 (3)	C18—C19—C20	121.1 (4)
C14—N2—C13	121.7 (4)	O3—C20—C15	126.4 (4)
C14—N2—Zn1	125.6 (3)	O3—C20—C19	115.9 (4)
C13—N2—Zn1	112.3 (3)	C15—C20—C19	117.7 (4)
O2—C1—C6	125.2 (4)	O5—S1—C21	107.4 (3)
O2—C1—C2	116.8 (4)	O5—S1—C22	105.0 (2)
C6—C1—C2	117.9 (4)	C21—S1—C22	96.9 (3)
O1—C2—C3	122.7 (4)	S1—C21—H21A	109.5
O1—C2—C1	117.5 (3)	S1—C21—H21B	109.5
C3—C2—C1	119.8 (4)	H21A—C21—H21B	109.5
C2—C3—C4	121.3 (4)	S1—C21—H21C	109.5
C2—C3—H3A	119.4	H21A—C21—H21C	109.5
C4—C3—H3A	119.4	H21B—C21—H21C	109.5
C5—C4—C3	119.3 (4)	S1—C22—H22A	109.5
C5—C4—H4A	120.3	S1—C22—H22B	109.5
C3—C4—H4A	120.3	H22A—C22—H22B	109.5
C4—C5—C6	121.7 (4)	S1—C22—H22C	109.5
C4—C5—H5A	119.2	H22A—C22—H22C	109.5
C6—C5—H5A	119.2	H22B—C22—H22C	109.5
C5—C6—C1	119.7 (4)	Zn1—O1W—H1W1	87 (4)
C5—C6—C7	115.7 (4)	Zn1—O1W—H2W1	114 (4)
C1—C6—C7	124.5 (4)	H1W1—O1W—H2W1	109 (3)
N1—C7—C6	126.3 (4)		
			
O4^i^—Zn2—O1—C2	−44.4 (3)	C6—C1—C2—C3	−5.4 (6)
O1^ii^—Zn2—O1—C2	112.0 (3)	O1—C2—C3—C4	−177.4 (4)
O3^i^—Zn2—O1—C2	−128.4 (2)	C1—C2—C3—C4	0.9 (6)
O2—Zn2—O1—C2	23.9 (2)	C2—C3—C4—C5	3.0 (7)
O4^i^—Zn2—O1—Zn2^i^	147.45 (15)	C3—C4—C5—C6	−2.3 (7)
O1^ii^—Zn2—O1—Zn2^i^	−56.06 (14)	C4—C5—C6—C1	−2.4 (7)
O3^i^—Zn2—O1—Zn2^i^	63.54 (17)	C4—C5—C6—C7	174.0 (4)
O2—Zn2—O1—Zn2^i^	−144.18 (17)	O2—C1—C6—C5	−177.2 (4)
O3—Zn1—O2—C1	130.5 (3)	C2—C1—C6—C5	6.2 (6)
O1W—Zn1—O2—C1	−120.3 (3)	O2—C1—C6—C7	6.7 (7)
N1—Zn1—O2—C1	−12.6 (3)	C2—C1—C6—C7	−169.9 (4)
N2—Zn1—O2—C1	33.0 (6)	C8—N1—C7—C6	176.2 (4)
O3—Zn1—O2—Zn2	−75.11 (16)	Zn1—N1—C7—C6	−13.8 (6)
O1W—Zn1—O2—Zn2	34.05 (18)	C5—C6—C7—N1	−177.3 (4)
N1—Zn1—O2—Zn2	141.76 (17)	C1—C6—C7—N1	−1.1 (7)
N2—Zn1—O2—Zn2	−172.6 (3)	C7—N1—C8—C9	−28.3 (7)
O4^i^—Zn2—O2—C1	107.0 (3)	Zn1—N1—C8—C9	160.6 (4)
O1^ii^—Zn2—O2—C1	−135.8 (2)	C7—N1—C8—C13	154.6 (4)
O1—Zn2—O2—C1	−25.5 (2)	Zn1—N1—C8—C13	−16.5 (5)
O3^i^—Zn2—O2—C1	65.3 (3)	C13—C8—C9—C10	−1.9 (7)
O4^i^—Zn2—O2—Zn1	−51.73 (17)	N1—C8—C9—C10	−179.0 (4)
O1^ii^—Zn2—O2—Zn1	65.53 (16)	C8—C9—C10—C11	1.6 (8)
O1—Zn2—O2—Zn1	175.75 (18)	C9—C10—C11—C12	0.2 (8)
O3^i^—Zn2—O2—Zn1	−93.4 (3)	C10—C11—C12—C13	−1.8 (7)
O1W—Zn1—O3—C20	87.5 (3)	C11—C12—C13—C8	1.5 (7)
O2—Zn1—O3—C20	−170.2 (3)	C11—C12—C13—N2	−177.0 (4)
N1—Zn1—O3—C20	−78.4 (4)	C9—C8—C13—C12	0.4 (7)
N2—Zn1—O3—C20	−8.4 (3)	N1—C8—C13—C12	177.7 (4)
O1W—Zn1—O3—Zn2^ii^	−95.24 (18)	C9—C8—C13—N2	179.0 (4)
O2—Zn1—O3—Zn2^ii^	7.07 (17)	N1—C8—C13—N2	−3.7 (6)
N1—Zn1—O3—Zn2^ii^	98.8 (2)	C14—N2—C13—C12	26.1 (7)
N2—Zn1—O3—Zn2^ii^	168.82 (18)	Zn1—N2—C13—C12	−159.8 (4)
O3—Zn1—N1—C7	−74.8 (4)	C14—N2—C13—C8	−152.4 (4)
O1W—Zn1—N1—C7	119.1 (3)	Zn1—N2—C13—C8	21.7 (5)
O2—Zn1—N1—C7	17.3 (4)	C13—N2—C14—C15	174.5 (4)
N2—Zn1—N1—C7	−149.3 (4)	Zn1—N2—C14—C15	1.3 (7)
O3—Zn1—N1—C8	95.8 (3)	N2—C14—C15—C20	−9.3 (8)
O1W—Zn1—N1—C8	−70.2 (3)	N2—C14—C15—C16	177.2 (4)
O2—Zn1—N1—C8	−172.0 (3)	C20—C15—C16—C17	0.7 (7)
N2—Zn1—N1—C8	21.4 (3)	C14—C15—C16—C17	174.7 (4)
O3—Zn1—N2—C14	5.9 (4)	C15—C16—C17—C18	−1.4 (7)
O1W—Zn1—N2—C14	−102.8 (4)	C16—C17—C18—C19	0.6 (7)
O2—Zn1—N2—C14	103.4 (5)	Zn2^ii^—O4—C19—C18	174.6 (3)
N1—Zn1—N2—C14	150.6 (4)	Zn2^ii^—O4—C19—C20	−5.1 (5)
O3—Zn1—N2—C13	−167.9 (3)	C17—C18—C19—O4	−178.9 (4)
O1W—Zn1—N2—C13	83.4 (3)	C17—C18—C19—C20	0.9 (7)
O2—Zn1—N2—C13	−70.4 (5)	Zn1—O3—C20—C15	4.3 (6)
N1—Zn1—N2—C13	−23.2 (3)	Zn2^ii^—O3—C20—C15	−173.3 (3)
Zn1—O2—C1—C6	3.8 (6)	Zn1—O3—C20—C19	−176.8 (3)
Zn2—O2—C1—C6	−153.8 (3)	Zn2^ii^—O3—C20—C19	5.7 (4)
Zn1—O2—C1—C2	−179.5 (3)	C16—C15—C20—O3	179.7 (4)
Zn2—O2—C1—C2	22.8 (4)	C14—C15—C20—O3	6.4 (7)
Zn2^i^—O1—C2—C3	−33.8 (5)	C16—C15—C20—C19	0.7 (6)
Zn2—O1—C2—C3	158.5 (3)	C14—C15—C20—C19	−172.5 (4)
Zn2^i^—O1—C2—C1	147.9 (3)	O4—C19—C20—O3	−0.8 (6)
Zn2—O1—C2—C1	−19.8 (4)	C18—C19—C20—O3	179.4 (4)
O2—C1—C2—O1	−4.0 (5)	O4—C19—C20—C15	178.2 (4)
C6—C1—C2—O1	173.0 (3)	C18—C19—C20—C15	−1.5 (6)
O2—C1—C2—C3	177.7 (4)		

Symmetry codes: (i) *y*, −*x*+1/2, −*z*+1/2; (ii) −*y*+1/2, *x*, −*z*+1/2.

### Hydrogen-bond geometry (Å, °)

**Table:**

*D*—H···*A*	*D*—H	H···*A*	*D*···*A*	*D*—H···*A*
O1W—H1W1···O4^i^	0.84 (4)	1.72 (4)	2.535 (4)	164 (5)
O2W—H1W2···O5	0.85 (8)	2.28 (9)	3.032 (4)	147 (4)
O1W—H2W1···O5	0.83 (4)	1.95 (4)	2.772 (5)	174 (4)
C3—H3A···O3^iii^	0.93	2.57	3.271 (5)	132
C21—H21C···O4^iv^	0.96	2.52	3.454 (7)	165
C21—H21B···Cg1^v^	0.96	2.81	3.475 (6)	127

Symmetry codes: (i) *y*, −*x*+1/2, −*z*+1/2;  (iii) −*x*+1/2, −*y*+1/2, *z*; (iv) −*y*+1, *x*+1/2, *z*−1/2; (v) −*x*+1, −*y*+1, −*z*.
